# Development of Alzheimer disease in old major depressive patients based upon their health status: A retrospective study in Taiwan

**DOI:** 10.1097/MD.0000000000015527

**Published:** 2019-05-17

**Authors:** Ping Tao, Szu-Nian Yang, Yu-Chi Tung, Ming-Chin Yang

**Affiliations:** a Department of Medical Affair and Planning, Taipei Veterans General Hospital; b Institute of Health Policy and Management, College of Public Health, National Taiwan University; c Superintendent Office, Tri-Service General Hospital Beitou Branch; d Institute of Health and Welfare Policy, National Yang-Ming University; e Department of Psychiatry, National Defense Medical Center, Taipei, Taiwan.

**Keywords:** Alzheimer disease, generalized estimating equation model, health status, major depressive disorder, survival analysis

## Abstract

This study is conducted to investigate the association between major depressive disorder and the subsequent development of Alzheimer disease (AD) in elderly patients with different health statuses using Taiwan's National Health Insurance Research Database (NHIRD).

A retrospective cohort study was performed on subjects over 65 years old from 2002 to 2006 using a random sampling from the 1 million beneficiaries enrolled in the NHI. Patients who were diagnosed with major depressive disorder were selected as the case group. Subjects in the control group were selected from elderly patients who did not have depression during the study period by matching age, sex, and index date of depression with subjects in the case group using a ratio of 1:4 (case:control). Both groups of patients were checked annually over a period of 7 years to observe whether they subsequently developed AD.

A total of 1776 subjects were included in the case group while 7104 subjects were in the control group. After the follow-up period, 59 patients (3.3%) with depression developed AD while 96 patients (1.4%) without depression developed AD. The Kaplan–Meier curves showed that the incidence rate of AD in both groups varied significantly depending on different health statuses (log-rank *P* < .001). Results of the generalized estimating equation model found that patients with depression (hazard ratio [HR] = 1.898; 95% confidence interval [CI] = 1.451–2.438), very severe health status (HR = 1.630; 95% CI = 1.220–2.177), or artery diseases (HR = 1.692; 95% CI = 1.108–2.584) were at a higher risk of developing AD than other groups.

The association between major depressive disorder and the later development of AD varied depending on the health statuses of elderly patients. Clinicians should exercise caution when diagnosing and treating underlying diseases in elderly depressed patients, and then attempt to improve their health status to reduce the incidence rate of subsequent AD development.

## 1 Introduction

The growth of elderly populations worldwide has increased attention to the early detection and treatment of age-related diseases, such as dementia. The number of dementia patients around the world was 47.5 million in 2016 and is expected to exceed 135.5 million by 2050.^[1,2]^ Alzheimer disease (AD) accounts for approximately 50% to 60% of all dementia patients. As the incidence rate of AD has increased year after year, the welfare and healthcare systems in many countries have incurred high costs.^[3–5]^ The World Health Organization has urged governments of developed countries to allocate resources to the treatment of patients with AD as a priority in national health policies.

Among elderly patients, depression is common and the prevalence of major depressive disorder in aged adults is approximately 12.9% to 21.7% worldwide. Depression, cancer, and AIDS are 3 major diseases that require particular attention in developed countries due to their high incidence rates and disease burden. In particular, depression results in huge social and economic loss.^[6]^ Older adults living in cities have 1.4 times the risk of developing depression than those living in rural areas. Moreover, women overall have twice the risk of suffering depression than men.^[7]^ Therefore, identifying depressive symptoms and effective treatments in elderly patients is of critical importance.

Precisely diagnosing whether older adults have depression or AD is often difficult because depression and AD share similar clinical symptoms in the early stages, such as poor memory, social withdrawal, apathy, and mild cognitive impairment.^[8]^ Previous studies have reported that elderly patients who are diagnosed with AD have a higher probability of suffering depression concurrently; however, these studies only showed that depression and AD are correlated and did not further investigate the potential of a causal relationship between these 2 diseases. In recent years, studies have implied that one's health status could be a moderator affecting development of diseases among elderly patients,^[9]^ especially for those who suffer from single or multiple chronic diseases. Therefore, investigating the influence of health status on the association between depression and AD in elderly patients is crucial. As a number of previous studies on the relationship between depression and AD used small samples from within specific institutions, the translation of these findings to the general population is limited.^[8,10,11]^ Examining the relationship between depression and AD based on population data sources is therefore necessary.^[10]^ The current study used subjects drawn from Taiwan's National Health Insurance Research Database (NHIRD) to examine the association between depression and AD among elderly patients with different health statuses. Results are discussed in the light of recommendations for improvements to clinical practice and health policy for geriatric patients with AD.

## 2 Methods

### 2.1 Data source

This retrospective cohort study used the 2000 to 2013 1 million beneficiary version of the NHIRD as its data source. The NHIRD has collected the demographic data of every beneficiary of the National Health Insurance (NHI) in Taiwan since 1995, including sex, age, diagnoses, and procedural codes in the International Classification of Disease, 9th version, Clinical Modification (ICD-9-CM), and itemized medical expenses for all types of medications, examinations, tests, therapies, and treatments. The ICD-9-CM codes were confirmed by computer programs and medical professionals to ensure satisfactory reliability and validity. Annually, the National Health Research Institute (NHRI) of Taiwan randomly selects 1 million beneficiaries to represent all insured Taiwanese to create the 1 million beneficiary version of the database and releases this to research organizations. This database has been used as the data source for many similar studies and all identifying data of subjects are replaced by encrypted numbers. This study was exempted from the Institutional Review Board (IRB).

### 2.2 Research samples

**Figure 1:**
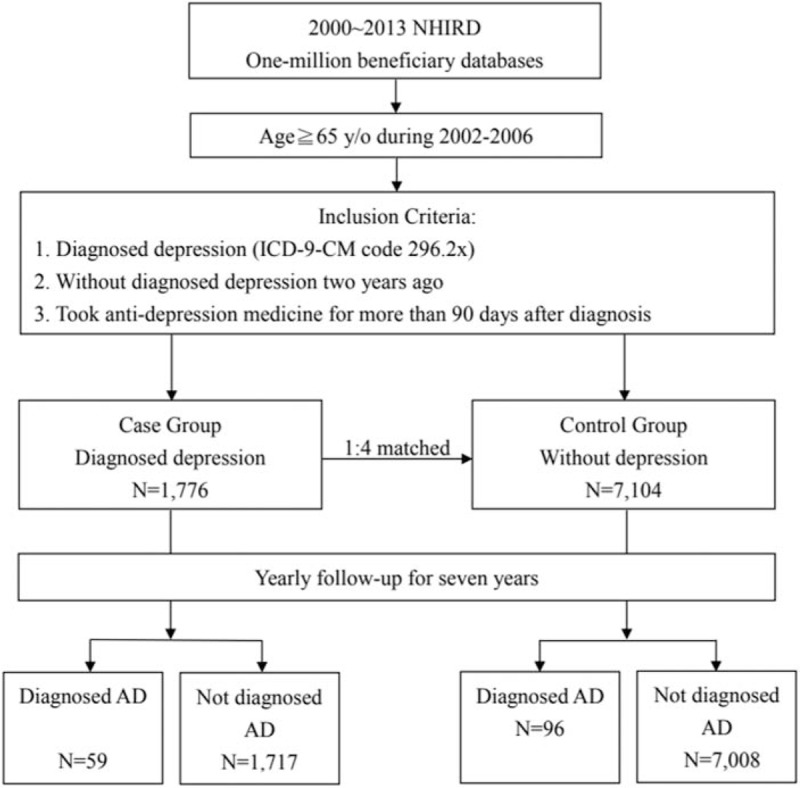
Process of selecting subjects from National Health Insurance Research Database (NHIRD).

Subjects in the case group were selected from patients aged 65 years old and over who were diagnosed for the 1st time with major depressive disorder (ICD-9-CM code 296.2x at least twice in 6 consecutive months) between 2002 and 2006, and prescribed antidepressant medication for 90 days and over within 6 months following the initial diagnosis. Subjects who were diagnosed with major depressive disorder 2 years before reaching 65 years old or who died within the following 7 years were excluded. Subjects in the control group were randomly selected by matching age, sex, and index date of depression (date when major depressive disorder was confirmed ±3 months) with the ones in the case group in a ratio of 1:4 (case:control). Subjects in the control group did not die within the 7-year period (Fig. 1).

### 2.3 Research variables

The major independent variable in this study was whether a patient had major depressive disorder. Another independent variable was health status, which was estimated based on the subject's annual utilization data by applying the adjusted clinical group (ACG) system developed by Johns Hopkins University. The ACG system calculates each subject's burden of morbidity, in terms of ACG's unscaled weights of the year, by analyzing the amount of health resources consumed in association with diagnoses and treatments of each utilization.^[12]^ The ACG system further divides burden of morbidity into 5 grades from healthy to very severe. This system has been widely adopted in studies on health status and its reliability and validity are widely accepted.

The dependent variable was whether a patient developed AD following diagnosis of major depressive disorder. Control variables included sex, age (while enrolled in the study), and comorbidities of the subjects. Comorbidities included coronary heart disease, ischemic heart disease, artery disease, arrhythmia, hypertension, diabetes, and chronic kidney disease.

### 2.4 Statistical analysis

The SAS 9.4 was used for statistical analysis. Numbers and percentages were used to describe categorical variables; mean and standard deviation were used to describe continuous variables. A Chi-square test was performed to examine the statistical association between 2 groups and a Fisher exact test was performed if an expected value for any categorical variable was <5. For the continuous variables, independent samples *t* tests were conducted to examine whether the mean difference for the 2 groups was significant. Kaplan–Meier curves and log-rank tests were performed to examine significant differences in developing AD between 2 groups. The incidence and hazard ratios of both groups were calculated with different health statuses. A generalized estimating equation (GEE) model was then used to assess the influence of depression on developing AD after controlling for each subject's health status and comorbidities during the 7-year period. Significance level (*α*) was set at 0.05.

## 3 Results

### 3.1 Sample characteristics

A total number of 8880 subjects were included in this study, and 59 out of the 1776 subjects in the case group acquired AD during the study period (Table 1). Subjects in the case group had a higher incidence rate of AD (3.3%) and poorer health status than those in the control group (very severe subjects accounted for 69.0% in the case group and 43.8% in the control group). The case group also had significantly more comorbidities: hypertension (81.0% vs 71.2%), type-2 diabetes (46.2% vs 34.8%), chronic kidney disease (15.6% vs 10.9%), artery disease (62.1% vs 44.1%), arrhythmia (37.7% vs 23.6%), coronary heart disease (7.7% vs 4.5%), and ischemic heart disease (39.5% vs 26.4%). Furthermore, subjects in the case group had a shorter survival time following development of AD than subjects in the control group (6.87 vs 6.96 years, *P* < .001).

**Table 1:**
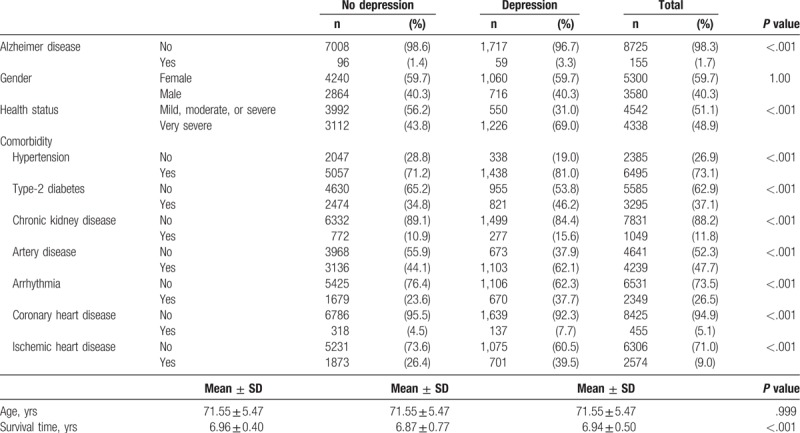
Baseline characteristics for subjects in study (n = 8880).

### 3.2 Risk of incidence in Alzheimer disease

Table 2 shows the risk of incidence in acquiring AD between the 2 groups. The incidence rate of AD for the case group was 0.96 per 10^3^ person-year (95% confidence interval [CI] = 0.73–1.22 per 10^3^ person-year) and the hazard ratio (HR) was 2.49 (95% CI = 1.80–3.45). The adjusted HR was 2.21 (95% CI = 1.57–3.11), which was also significantly higher than that of the control group. In addition, the incidence rate of AD for the case group with mild, moderate, or severe health statuses was 0.54 per 10^3^ person-year (95% CI = 0.32–0.78 per 10^3^ person-year), but for the control group, it was 1.39 per 10^3^ person-year (95% CI = 1.02–1.85 per 10^3^ person-year). The HR and adjusted HR for the case group in mild, moderate, or severe health status was 2.84 (95% CI = 1.62–4.97) and 2.67 (95% CI = 1.50–4.74), respectively. The incidence rate of AD for the case group with very severe health status was 1.40 per 10^3^ person-year (95% CI = 1.06–1.82 per 10^3^ person-year), but for the control group, it was 1.73 per 10^3^ person-year (95% CI = 1.30–2.24 per 10^3^ person-year). The HR and adjusted HR for the case group with very severe health status was 2.08 (95% CI = 1.38–3.12) and 2.06 (95% CI = 1.36–3.12), respectively.

**Table 2:**
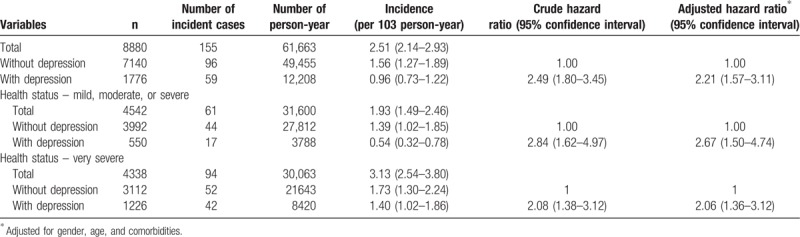
Risk of incidence in Alzheimer disease.

**Figure 2:**
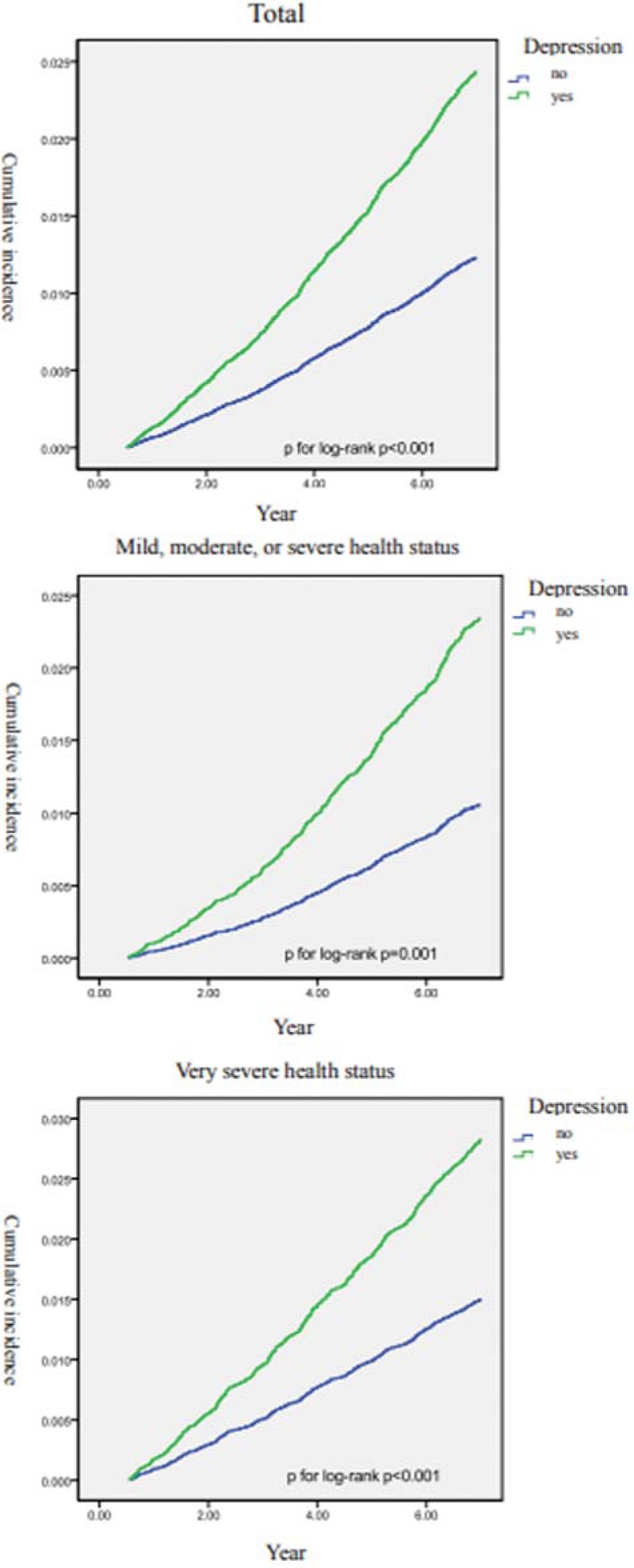
Cumulative incidence curves of Alzheimer disease (AD) from all patients, from patients with mild, moderate, or severe morbidity status, and from very severe morbidity status-log-rank *P* of all 3 graphs significant at *P* < .05).

Figure 2 is the Kaplan–Meier curves of the 2 groups without accounting for health status. These reveal that the case group had a higher incidence rate of AD than the control group. A similar finding was observed even after accounting for various health statuses. The result of the log-rank test showed that the survival rates for the 2 groups differed significantly (*P* < .001).

### 3.3 Association between major depressive disorder and Alzheimer disease

The GEE models on patients with major depressive disorder and AD are exhibited in Table 3. Independent variables influencing the development of AD included age, severity of health status, artery disease, and major depressive disorder. For every yearly increase in age, the patients had 1.055 times the risk of developing AD (95% CI = 1.035–1.075). Additionally, patients with very severe health statuses had 1.630 times the risk of developing AD than those with mild to severe health statuses (95% CI = 1.220–2.177). Subjects suffering from artery disease had 1.692 times the risk of developing AD than those without artery disease (95% CI = 1.108–2.584). Finally, the case group diagnosed with major depressive disorder had 1.898 times the risk of developing AD than the control group (95% CI = 1.451–2.483) (Table 4).

**Table 3:**
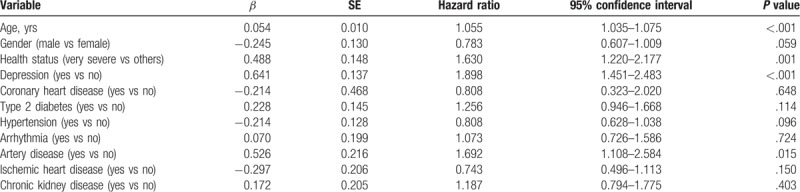
Generalized estimating equation (GEE) models for patients with depression and Alzheimer disease (n = 8880).

**Table 4:**
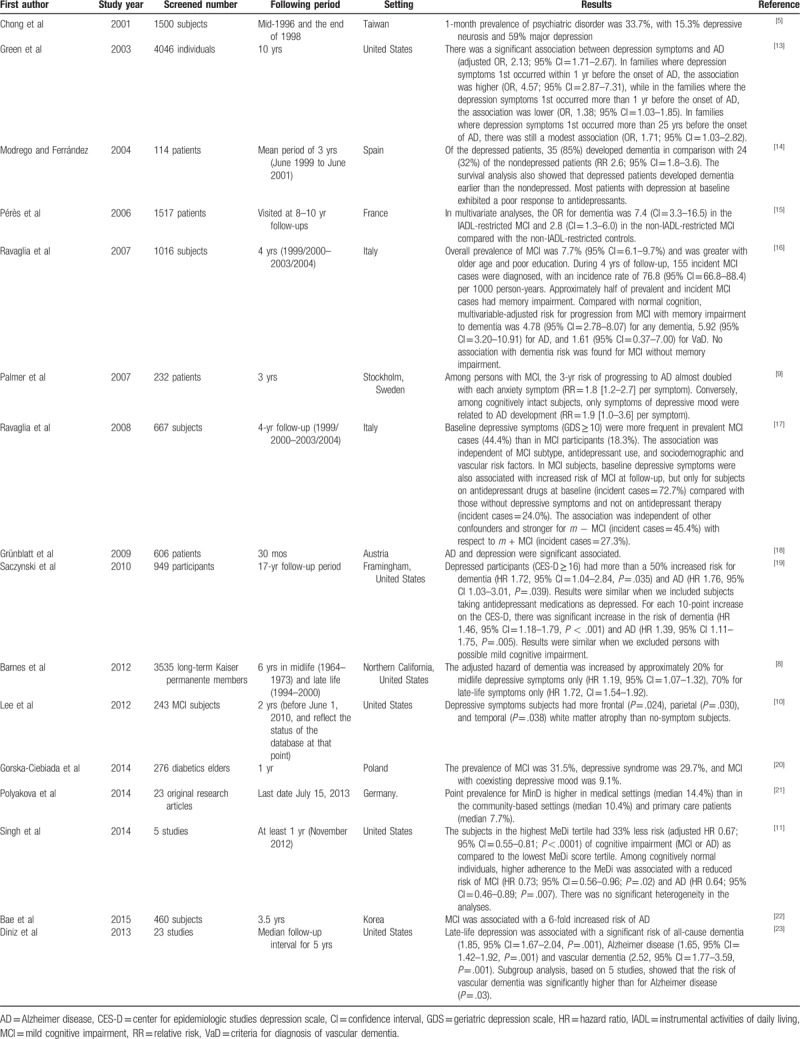
Study results of development of Alzheimer disease form major depression patients in various populations.

## 4 Discussion

### 4.1 Clinical implications

Although a number of previous studies have found that depressive elderly patients have a higher risk of developing AD than those without depression, the degree of association between depression and AD varied across studies. Our study identified an association between different health statuses of subjects diagnosed with depression, and thereafter the development of AD, irrespective of the age, sex, and comorbidities of these patients. Since this study was a longitudinal and retrospective cohort study with 7 consecutive years of follow-up and defined all subjects with single-episode major depressive disorder, the results of the association between depression and AD must be interpreted with caution. The results showed that depressive elderly patients had a higher AD incidence rate (0.96 per 10^3^ person-year; 95% CI = 0.73–1.22 per 10^3^ person-year) and an HR of 2.49 (95% CI = 1.80–3.45) relative to those without depression. The adjusted HR to develop subsequent AD was 2.21 (95% CI = 1.57–3.11). In addition, this study used the ACG system to quantify health status so that we could investigate the degree of association between depression and subsequent AD, and health status objectively. The variables were often measured by questionnaires in the past. As the health status of all subjects was calculated for 7 years, the significance of our study differs from previous studies because the utilization data used to calculate health status each year and for every subject were complete. The causal relationship between AD and depression in elderly adults could not be determined in previous studies because often the length of observation was too short.^[14,16,17,24]^

Many previous studies investigating the relationship between depression in elderly patients and subsequent development of AD used only clinical data including MRI images. However, a lack of longitudinal utilization data for observation prevented the identification of a causal relationship.^[9]^ According to Saczynski et al, inconsistent results from those studies that investigated the influence of elderly depression on dementia were possibly caused by the short time periods of subsequent follow-up.^[19]^ Additionally, problems in early studies arose due to the short time between the confirmation of depression and acquisition of AD.^[19]^ Our study followed its subjects for 7 years and found that there was a higher possibility of developing AD in elderly patients with depression than those without depression; therefore, clinicians need to pay special attention to this association while treating elderly patients suffering from depression.

The vast majority of AD cases have a late onset (usually ≥65 years of age). This disease is rare among younger individuals.^[25]^ Age is recognized as one of the major risk factors for the development of AD. Our study also demonstrated the same result by both univariate analysis and the GEE model. This suggests that older age is symbolic of an indicator for AD. From the clinical viewpoint, age at onset of AD is often a very difficult parameter to record with precision when assessed retrospectively. The ability to delay the age at onset of this disease through preventive or therapeutic approaches would have significant benefits. However, few therapies have been successful in achieving this important goal.^[26]^

It was strongly linked to the health status of the elderly and development of AD after adjustment for confounding factors in this study. Previous study indicated that elderly people's need to adopt lifestyle modifications for their health. However, lifestyle changes are not made easily.^[27]^ Functional status and health status are both highly related to the quality of life, and especially so for the elderly.^[28]^ To decrease the progression of AD, it is essential that healthcare services be easily accessible and address the elderly population's increasing needs for health management, risk reduction, help adopting healthy lifestyles, and improving personal quality of life.

Another association found in this study was the influence of artery diseases on elderly patients with depression and subsequent AD. Artery diseases have been identified as a comorbidity or risk factor causing AD and were well-documented in previous studies. A subtype of late-onset depression is vascular depression. Some vascular risk factors (e.g., hypertension, diabetes, and heart disease) cause neural pathways responsible for regulating emotion to exhibit vascular lesions. This leads to the destruction of emotional regulation and results in vascular depression.^[29]^ Despite this, we found that only artery disease had a significant impact on AD. Therefore, clinicians must be careful to distinguish between depression and vascular depression because the latter can cause cognitive dysfunction similar to AD. In particular, patients with prominent executive dysfunction have a relatively high possibility of being diagnosed with dementia.^[30]^ As a result, artery disease could be treated as a comorbidity which moderates depression but not vascular depression or AD.

In this study, the association between depression and AD was the way in which the diagnosis of AD was also confirmed. Since medication for AD is expensive compared with those used in other chronic diseases, the NHI bureau requests that physicians exclude depression before confirming a diagnosis of AD. Nonetheless, previous studies have found that many patients exhibit mild cognitive impairment (MCI) before the confirmation of AD; therefore, the evidence of AD warrants discussion.^[31]^ Panza et al found that the incidence and prevalence of depression varied considerably in some MCI studies mainly because the diagnostic conditions, sampling, and assessment methods for MCI and AD were similar, but the prevalence of MCI and AD also differed substantially.^[24]^ Some studies considered symptoms of MCI as evidence of early-stage AD; that is, confirmation of AD included preexisting MCI.^[24]^ Polyakova et al agreed that inconsistent research methods not only resulted in considerably different prevalence rates of depression among patients with AD but also led to difficulties interpreting the results due to the confirmation of AD.^[21]^ The diagnosis of MCI, though, is rare in the NHIRD, and for this reason, we did not include MCI as a variable.

There was no evidence that sex had any influence on the development of AD in our study. The risk of developing AD in depressed patients increased by 1.055 times compared to those without depression for every year older. The relationship between depression and AD confirmed the findings of previous studies.^[15,16,20,22]^ Our study confirmed that health status, as derived by applying the ACG software to utilization data for 7 years, was a significant moderator of the correlation between depression and AD. Although comorbidities could be multicollinear in correlation to health status, a high level of multicollinearity for the derived data of health status was not necessary because the results of the ACG calculation of health status have been proven useful in studies of this kind.

### 4.2 Methodologic considerations

Our study has several potential limitations. Firstly, because we used a more conservative method to select subjects who had depression and/or AD by diagnosis and medication instead of by questionnaire, some subjects with depression or AD diagnosis/medication could have been excluded due to a lack of information. However, since the NHI in Taiwan is a compulsory insurance covering 99% of the total population of Taiwan, we believe that unidentified cases of elderly patients with both diseases are rare. Secondly, because the treatment of both depression and AD in Taiwan is expensive, the NHI bureau requests that physicians go through complex processes to confirm the incidence of both diseases. Therefore, the influence of depression on AD might be underestimated because of the high stringency in selecting subjects. Since over 99% of total population is insured by the NHI, the impact of uninsured subjects on our findings should be minimal. Thirdly, the data source of this study was the NHIRD which lacked relevant clinical variables such as laboratory data and pathology findings. A misclassification bias might have been present. Finally, due to the sex effect in this study was viewed as one of confounding factors when explore the association between major depressive disorder and the later development of AD, further studies should be conducted to analyses the potential for condition-related sex difference, because it was considered that such difference might underscore important implications for the understanding of the overall pathogenesis of the development of AD among the old major depressive patients.

## 5 Conclusion

In conclusion, the onset of AD is found in approximately one-third of the elderly patients with AD also suffering from depression. Symptoms related to cognitive functions are unclear; therefore, accurate diagnosis relies on the reliability of information provided by families or care-givers. Unfortunately, some patients’ families or care-givers also suffer from depression or other emotional problems while taking care of the patients. This may influence the information provided. Therefore, hospitalization often proves to be a more reliable method of diagnosis. Thus, different diagnostic codes should be devised for geriatric patients with depression and other depressive symptoms.

## Author contributions

Ping Tao, Szu-Nian Yang, Yu-Chi Tung, Ming-Chin Yang conducted the study and drafted the manuscript. Szu-Nian Yang and Yu-Chi Tung participated in the design of the study and performed statistical analyses. Ping Tao and Ming-Chin Yang conceived the study, and participated in its design and coordination. All of the authors read and approved the final manuscript.

**Conceptualization:** Yu-Chi Tung, Ming-Chin Yang.

**Formal analysis:** Szu-Nian Yang.

**Methodology:** Szu-Nian Yang.

**Supervision:** Ming-Chin Yang.

**Writing – original draft:** Ping Tao, Szu-Nian Yang, Yu-Chi Tung.

**Writing – review & editing:** Ping Tao, Ming-Chin Yang.
